# The relationship between *RRM1* gene polymorphisms and effectiveness of gemcitabine-based first-line chemotherapy in advanced NSCLC patient

**DOI:** 10.1007/s12094-015-1461-1

**Published:** 2015-12-09

**Authors:** R. Mlak, P. Krawczyk, M. Ciesielka, P. Kozioł, I. Homa, T. Powrózek, M. Prendecka, J. Milanowski, T. Małecka-Massalska

**Affiliations:** 1 Department of Human Physiology, Medical University of Lublin, Radziwiłłowska 11, 20-080 Lublin, Poland; 2 Department of Pneumology, Oncology and Allergology, Medical University of Lublin, Radziwiłłowska 11, 20-080 Lublin, Poland; 3 Department of Forensic Medicine, Medical University of Lublin, Radziwiłłowska 11, 20-080 Lublin, Poland

**Keywords:** RRM1, Chemotherapy, Gemcitabine, Platinum compounds, Non-small cell lung cancer

## Abstract

**Purpose:**

Chemotherapy with platinum compounds and gemcitabine is frequently used in first-line treatment of advanced non-small cell lung cancer (NSCLC) patients in which tyrosine kinase inhibitors (EGFR or ALK) cannot be administered. Unfortunately, less than half of the patients achieve the benefit from chemotherapy. Gemcitabine is an analog of deoxycytidine (pyrimidine antimetabolite) with antitumor activity. The excess of deoxycytidine synthesized by RRM1 enzyme activity may be a cause of competitive displacement of gemcitabine, which reduces the efficacy of this cytostatic. The aim of this study was to determine the association between single nucleotide polymorphisms (SNPs) of the *RRM1* promoter (−37C>A, −524C>T) and the effectiveness of first-line chemotherapy based on platinum compounds and gemcitabine in NSCLC patients.

**Patients and methods:**

SNPs were determined by SNaPshot PCR^®^ in DNA isolated from peripheral blood of 91 NSCLC patients.

**Results:**

The median progression-free survival (PFS) was significantly longer in carriers of AA (−37C>A) as well as CC (−524C>T) genotype of *RRM1* compared to patients with other genotypes (10.5 vs 3.5 months, *p* = 0.0437; HR = 2.17, 95 % CI 1.02–4.62 and 10.5 vs 3.5 months, *p* = 0.0343; HR = 2.12, 95 % CI 1.06–4.27). In addition, the CC genotype carriers (−37C>A) showed a significant increase in the risk of shortening overall survival (OS) in comparison to patients with AA or AC genotypes (9.5 vs 18 months, *p* = 0.0193; HR = 2.13, 95 % CI 1.13–4.03).

**Conclusions:**

Presence of rare AA (−37C>A) and CC (−524C>T) genotypes of the *RRM1* may be favorable predictive factors for chemotherapy with platinum compounds and gemcitabine in NSCLC patients.

## Introduction

Non-small cell lung cancer (NSCLC, representing approx. 85 % of lung cancer cases) is still the most common cause of death (1.6 million in 2012) due to malignancies in developed countries [1]. Despite the dynamic development of medicine, the chemotherapy based on platinum compounds (cis- or carboplatin) and third generation drugs (e.g. gemcitabine) still remains standard regimen of the first-line treatment of advanced NSCLC. After administration of standard first-line chemotherapy, the objective response rates (ORR) varies between 20 and 30 %. Chemotherapy provides slight prolongation of patients’ survival time (1.5 months compared to the best supportive care, BSC); however, its use is associated with occurrence of relevant toxicity. Median overall survival (OS) of patients treated by systemic therapy range from 6 to 12 months [2]. Identification of driver mutations or other gene alterations (e.g. activating mutations in the *EGFR* and *ALK* rearrangements), which are potential molecular targets, seems to be an important advancement in optimization and individualization of NSCLC therapy. These drugs significantly improved treatment outcomes (over 60 % ORR, progression free survival (PFS) prolonged to 10 months) with acceptable toxicity [3–6]. Only 10–30 % (*EGFR* mutations) or 3–7 % (*ALK* rearrangements) Caucasian patients with advanced non-squamous NSCLC have molecular alterations and should be treated by target therapy. The lack of identified molecular targets for squamous cell carcinoma treatment is the reason why majority of patients still receive standard chemotherapy [7–9]. On the other hand, genetic predisposition (e.g. gene polymorphisms or mRNA expression) may be used to selection for potentially the most effective treatment regimens, which can prolong the life of patients and improve its quality [10–12]. Among the molecular changes, potentially the highest impact on the efficacy of chemotherapy have alterations of genes, which coding proteins involved in drug metabolism. Gemcitabine is frequently used in the treatment of NSCLC, ovarian and pancreatic cancer. Its mechanism of action is based on the incorporation to nucleic acids, which consequently induce apoptosis. The excess of deoxycytidine, biosynthesized with the participation of RRM1 causes competitive displacement of gemcitabine, reducing the efficacy of this chemotherapeutic agent [13, 14]. Some researchers have demonstrated that in patients with NSCLC the expression or SNPs of *RRM1* may play prognostic and predictive role (e.g. for gemcitabine). The influence of single nucleotide polymorphisms (SNPs) on survival and the response to treatment with platinum compounds and gemcitabine in patients with advanced NSCLC are still not fully understood. Among the already known *RRM1* SNPs, −37C>A (rs12806698) and −524C>T (rs11030918) seem to have the greatest importance as potential predictors of treatment regimens based on gemcitabine in NSCLC patients [15–17].

The aim of this study was to determine the association between SNPs of *RRM1* promoter (−37C>A, −524C>T) and the effectiveness of chemotherapy based on platinum compounds and gemcitabine in patients with inoperable or advanced NSCLC.

## Materials and methods

The study was performed on 91 Caucasian patients with inoperable, locally advanced or metastatic NSCLC (IIIB and IV), treated from 2010 to 2013 at the Department of Pneumonology, Oncology and Allergology, Medical University of Lublin. NSCLC diagnosis was based on histopathological or cytological examination. In the first-line treatment all patients received standard chemotherapy, based on platinum compounds and gemcitabine. The stage of disease was evaluated according to the TNM classification (VII edition by UICC). The median number of cycles of first-line chemotherapy was 4 (range 2–6). Subsequent lines of therapy: second or third were used in 56.1 and 16.5 % of patients, respectively (Table 1). Response to treatment was evaluated by RECIST V1.1 (Response Evaluation Criteria in Solid Tumors). Adverse events were estimated by Common Toxicity Criteria for Adverse Events (CTCAE) V4.0.

**Table 1:** Patient characteristics

Variable	Study group (*n* = 91)
Sex	
Male	61 (67 %)
Female	30 (33 %)
Age (years)	
Median	62
Mean ± SD	62.5 ± 7.9
Range	38–78
Smoking status (pack-years)	
Median	30
Mean ± SD	31.4 ± 9.5
Non-smokers	5 (5.5 %)
Current smokers	65 (71.4 %)
Former Smokers	20 (22 %)
No data	1 (1.1 %)
Histopathological diagnosis	
Adenocarcinoma	46 (50.5 %)
Squamous cell carcinoma	14 (15.4 %)
Large cell carcinoma	16 (17.6 %)
NOS (not otherwise specified)	15 (16.5 %)
Stage of disease	
IIIB	28 (30.8 %)
IV	63 (69.2 %)
Performance status	
PS = 0	12 (13.2 %)
PS ≥ 1	79 (86.8 %)
Weight loss before CTH	
Yes	39 (42.9 %)
No	43 (47.2 %)
No data	9 (9.9 %)
Anemia before CTH	
Yes	59 (64.8 %)
No	32 (35.2 %)
Side effect after I line CTH	
Yes	59 (64.8 %)
No	24 (26.4 %)
No data	8 (8.8 %)
Subsequent lines of treatment	
Yes	51 (56.1 %)
No	40 (43.9 %)
Second-line CTH (monotherapy)	**51 (56.1** **%)**
ERL	12 (13.2 %)
PEM	26 (28.6 %)
DCX	13 (14.3 %)
Third-line CTH (monotherapy)	**15 (16.5** **%)**
ERL	5 (5.5 %)
PEM	6 (6.6 %)
DCX	4 (4.4 %)

*ERL* erlotinib, *DCX* docetaxel, *PEM* pemetrexed

**Fig. 1:**
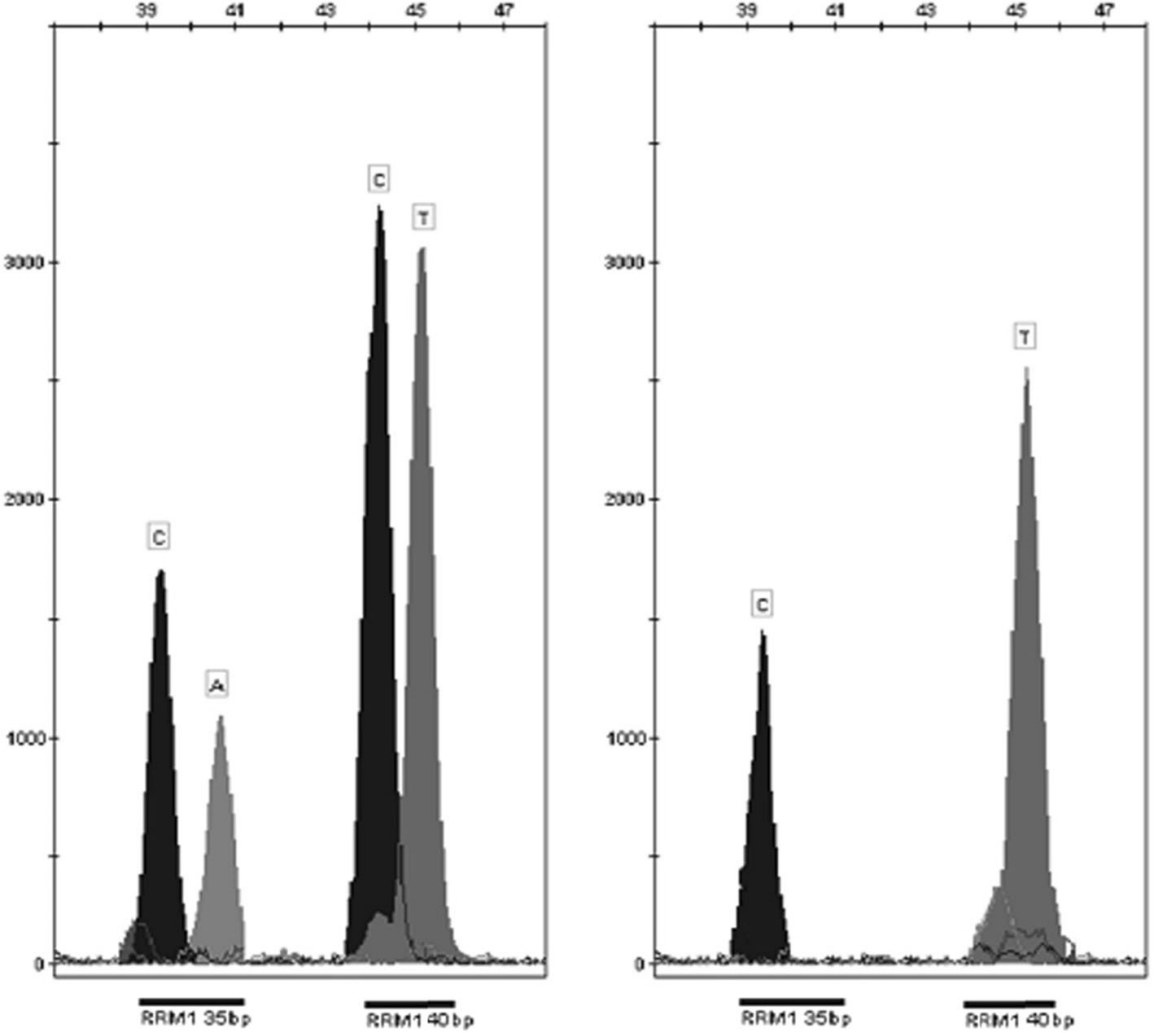
Example of genotyping results of *RRM1* gene obtained by capillary electrophoresis of the SNaPshot PCR products. From *left* (−37C>A and −524C>T respectively): AC and TT heterozygotes, CC and TT homozygotes

The isolation of DNA from peripheral blood leukocytes was performed using DNA Blood Mini Kit (Qiagen, Canada). Quality and quantity of extracted DNA were measured using a spectrophotometer BioPhotometer plus in cuvette equipped with UV/VIS filters (Eppendorf, Germany). Analysis of SNPs was conducted using the mini-sequencing technique (SNaPshot^®^ PCR). For the reaction, a set of ABI PRISM SNaPshot^®^ Multiplex (Life Technologies, USA) was used. An example of the results of genotyping is shown in Fig. 1.

### Statistical analysis

Statistical analysis was done using Statistica 10 (Statsoft, USA) and MedCalc 10 (MedCalc Software, Belgium). The results of *p* < 0.05 were considered to be statistically significant. Using the Chi-Square (*χ*^2^) balance of the Hardy–Weinberg (HW) equilibrium, associations between a series factors with the distribution of *RRM1* polymorphisms were calculated. The Kaplan–Meier method was used to draw a comparison curve evaluating the survival probability (PFS and OS). Cox regression model with a stepwise selection with minimum AIC factor (Akaike Information Criterion) was used to determine the influence of clinical and genetic factors on survival. The median survival in the groups was compared by the use of the *U*-Mann–Whitney test.

## Results

Distributions of genotypes in the *RRM1* (−37C>A, −524C>T) did not depend on factors such as gender, age, histological type, stage of disease, performance status or smoking status (Table 2). The distribution of genotypes within the SNP −524C>T, unlike the −37C>A, was in Hardy–Weinberg equilibrium (*p* = 0.0966, *χ*^2^ = 2.7612 for −524C > T and *p* = 0.0279, *χ*^2^ = 4.8336 for −37C>A). Genotypes AA, AC and CC of *RRM1* (−37C>A) occurred respectively in 5.7, 54.5 and 39.8 % of patients. Genotypes CC, CT and TT of *RRM1* (−524C>T) occurred, respectively, in 7.7, 52.7 and 39.6 % of patients. Due to the fact that studied SNPs are located in the same gene (on chromosome 11) using the online tool SNAP (Broad Institute, USA) and data from the International HapMap project assesses coupling allele linkage disequilibrium (LD), LD was calculated based on data from population-based genetic studies (Utah state residents whose ancestors came from the north-western regions of Europe–CEU). Analyzed pair of SNPs has not reached the correlation coefficient *R*^2^ > 0.8 so, we concluded that they do not create haplotypes and are not subject to the common inheritance.

**Table 2:** *RRM1* gene genotypes distribution according to demographic and clinical factors

Variable	*RRM1* (−37C>A)			*p*, *χ* ^2^	*RRM1* (−524C>T)			*p*, *χ* ^2^
	AA	AC	CC		CC	CT	TT	
	5 (5.7 %)	48 (54.5 %)	35 (39.8 %)		7 (7.7 %)	48 (52.7 %)	36 (39.6 %)	
Sex								
Male	2 (6.7 %)	16 (53.3 %)	12 (40 %)	0.9557	3 (10 %)	14 (46.7 %)	13 (43.3 %)	0.6755
Female	3 (5.2 %)	32 (55.2 %)	23 (39.6 %)	0.091	4 (6.6 %)	34 (55.7 %)	23 (37.7 %)	0.785
Age (years)								
<70	3 (4.2 %)	39 (54.9 %)	29 (40.9 %)	0.4751	6 (8.2 %)	40 (54.8 %)	27 (37 %)	0.5932
≥70	2 (11.8 %)	9 (52.9 %)	6 (35.3 %)	1.488	1 (5.6 %)	8 (44.4 %)	9 (50 %)	1.045
Smoking status								
Current smokers	4 (6.2 %)	35 (54.7 %)	25 (39.1 %)	0.5369	5 (7.7 %)	35 (53.8 %)	25 (38.5 %)	0.0505
Ex-smokers	–	10 (55.6 %)	8 (44.4 %)	3.126	–	10 (50 %)	10 (50 %)	9.464
Non-smokers	1 (20 %)	2 (40 %)	2 (40 %)		2 (40 %)	2 (40 %)	1 (20 %)	
Histopathological diagnosis								
Adenocarcinoma	3 (6.8 %)	22 (50 %)	19 (43.2 %)	0.6648	4 (8.7 %)	26 (56.5 %)	16 (34.8 %)	0.8393
Squamous cell carcinoma	–	8 (57.1 %)	6 (42.9 %)	4.088	–	7 (50 %)	7 (50 %)	2.752
Large cell carcinoma	1 (6.7 %)	11 (73.3 %)	3 (20 %)		2 (12.5 %)	7 (43.75 %)	7 (43.75 %)	
NOS NSCLC	1 (6.6 %)	7 (46.7 %)	7 (46.7 %)		1 (6.7 %)	8 (53.3 %)	6 (40 %)	
Stage of disease								
IIIB	3 (11.1 %)	16 (59.3 %)	8 (29.6 %)	0.2032	2 (7.1 %)	15 (53.6 %)	11 (39.3 %)	0.9891
IV	2 (3.3 %)	32 (52.4 %)	27 (44.3 %)	3.187	5 (7.9 %)	33 (52.4 %)	25 (39.7 %)	0.022
Performance status								
PS = 0	1 (8.3 %)	7 (58.3 %)	4 (33.3 %)	0.8381	1 (9.1 %)	5 (45.45 %)	5 (45.45 %)	0.9889
PS ≥ 1	4 (5.3 %)	41 (53.9 %)	31 (40.8 %)	0.353	6 (9.2 %)	28 (43.1 %)	31 (47.7 %)	0.022

### Response to chemotherapy

There were no cases of complete remission (CR) as a result of first-line chemotherapy with platinum compounds and gemcitabine. Control of the disease was observed in 54.9 % of patients, of which partial remission (PR) and stable disease (SD) occurred, respectively, in 17.6 and 37.3 % of patients. Disease progression (PD) was observed in 45.1 % of patients. Patients with squamous cell carcinoma or those who develop anemia before chemotherapy had a significantly lower chance of disease control (*p* = 0.0392, OR = 0.27; *p* = 0.0189, OR = 0.33, respectively) when compared to other patients. Moreover, in patients with poor performance status (PS = 1) the risk of PD (*p* = 0.0495, OR = 4.87) was higher. There were no statistically significant differences in response according to other demographic and clinical factors. Both SNPs: −37C>A and −524C>T of *RRM1* did not affect significantly the possibility of responses to treatment. In logistic regression analysis (including: sex, age, histopathological diagnosis, stage of disease, PS and SNPs of *RRM1*; overall fit of the model: *χ*^2^ = 22.45, *p* = 0.0021) only PS (*p* = 0.0002, OR = 0.0849 95 % CI 0.02–0.31) had an independent influence on ORR. Effect of SNPs of *RRM1* was insignificant, however, the CC genotype (−37C>A) show a trend towards significance (*p* = 0.0655).

### Progression-free survival

The median PFS of whole group of patients was 4 months. In patients who were diagnosed with adenocarcinoma or non-squamous cell carcinoma compared to squamous cell carcinoma patients a significantly lower risk of shortening PFS was noticed (respectively, 6 vs 3 months, *p* = 0.0456, HR = 0.61, 95 % CI 0.37–0.99 and 4.5 vs 2 months, *p* = 0.0140, HR = 0.34, 95 % CI 0.14–0.80). In patients with IIIB stage of NSCLC and patients without anemia compared to other patients, the risk of shortening of PFS was significantly lower (respectively, 7 vs 3, *p* = 0.0094, HR = 0.52, 95 % CI 0.32–0.85; 6.5 vs 3 months, *p* = 0.0154, HR = 0.54, 95 % CI 0.33–0.89). Other factors did not affect PFS significantly.

**Fig. 2:**
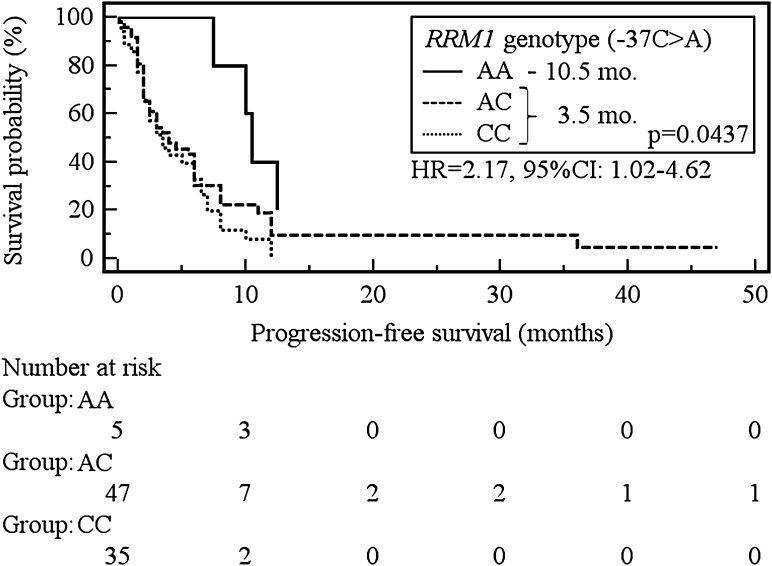
The probability of progression-free survival change depending on *RRM1* genotype (−37C>A)

**Fig. 3:**
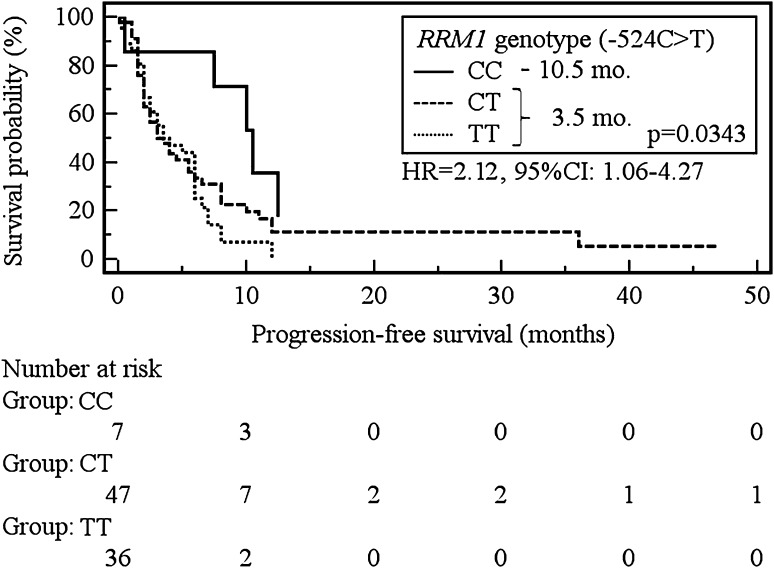
The probability of progression-free survival change depending on *RRM1* genotype (−524C>T)

Carriers of the C allele of the *RRM1* (−37C>A) showed a significant increase in the risk of PFS shortening in comparison to patients with the AA genotype (3.5 vs 10.5 months, *p* = 0.0437; HR = 2.17, 95 % CI 1.02–4.62, Fig. 2). Similarly, the presence of the T allele of *RRM1* (−524C>T) was associated with a significant risk of shortening of PFS when confronted with the CC genotype carriers (3.5 vs 10.5 months, *p* = 0.0437; HR = 2.12, 95 % CI 1.06–4.27, Fig. 3).

In a Cox multivariate logistic regression analysis, poor (PS = 1) performance status (*p* = 0.0117, HR = 2.75, 95 % CI 1.26–6.02) and anemia prior to treatment (*p* = 0.0128, HR = 2.67, 95 % CI 1.24–5.74) were independently responsible for shortening of PFS in patients treated with chemotherapy using platinum compounds and gemcitabine (overall fit of the model: *p* = 0.0271, *χ*^2^ = 24.466) (Table 3).

**Table 3:** Effect of demographic, clinical and genetic factors on overall response rates, progression-free survival and overall survival in the study group

Variable	PD 41 (45.1 %)	SD, PR 50 (54.9 %)	*p*, OR 95 % CI	Median PFS (mo.)	*p, χ* ^2^	HR 95 % CI	Median OS (mo.)	*p, χ* ^2^	HR (95 % CI)
Sex									
Male	31 (50.8 %)	30 (49.2 %)	0.1179, 0.4839	4	0.8653	0.9560	13	0.7280	1.1177
Female	10 (33.3 %)	20 (66.7 %)	0.1948–1.2022	6	0.0288	0.5684–1.6079	11	0.1210	0.5971–2.0923
Age (years)									
≤70	34 (46.6 %)	39 (53.4 %)	0.5581, 0.7299	3.56	0.6344	0.8719	11.5	0.7812	0.9061
>70	7 (38.9 %)	11 (61.1 %)	0.2546–2.0929		0.2262	0.4956–1.5339	16.5	0.0772	0.4519–1.8169
Smoking status									
Smokers	39 (45.9 %)	46 (54.1 %)	0.7978, 0.7863	47.5	0.5347	0.7441	11.5	0.8851	0.9187
Non-smokers	2 (40 %)	3 (60 %)	0.125–4.9481		0.3854	0.2926–1.8921	18.5	0.0209	0.2909-2.9012
Histopathological diagnosis									
Adenocarcinoma	18 (40 %)	27 (60 %)	–	6	0.1356	–	12	0.6073	–
Squamous cell carcinoma	10 (71.4 %)	4 (28.6 %)		2	5.5517		6.5	1.8352	
Large cell carcinoma	8 (47.1 %)	9 (52.9 %)		4			13		
NOS NSCLC	5 (33.3 %)	10 (66.7 %)		3.5			9.5		
Adenocarcinoma	18 (39.1 %)	28 (60.9 %)	0.2520, 1.6263	6	0.0456	0.6074	12	0.4094	0.7802
Other	23 (51.1 %)	22 (48.9 %)	0.7077–3.7371	3	3.9962	0.3725–0.9903	11.5	0.6805	0.4326–1.4071
Squamous cell carcinoma	10 (71.4 %)	4 (28.6 %)	0.0392, 0.2696	2	0.0140	0.3399	6.5	0.1807	0.5301
Non-squamous cell carcinoma	31 (40.3 %)	46 (59.7 %)	0.0776–0.9369	4.5	6.0383	0.1437–0.8038	12	1.7916	0.2093–1.3427
Large cell carcinoma	8 (50 %)	8 (50 %)	0.6619, 0.7857	3.754	0.4972	0.8030	13	0.9921	1.0036
Other	33 (44 %)	42 (56 %)	0.2666–2,3157		0.4608	0.4263–1.5128	11.5	0.0001	0.4928–2.0439
Stage of disease									
IIIB	10 (35.7 %)	18 (64.3 %)	0.2348, 1.7438	7	0.0094	0.5212	18	0.0701	0.5687
IV	31 (49.2 %)	32 (50.8 %)	0.6968–4.3641	3	6.7519	0.3188–0.8520	10	3.2800	0.3088–1.0475
Performance status									
PS = 0	2 (16.7 %)	10 (83.3 %)	0.0495, 4.8750	7.5	0.2694	0.7024	21	0.0700	0.5124
PS ≥1	39 (49.4 %)	40 (50.6 %)	1.0031–23.691	3.5	1.2197	0.3752–1.3148	11	3.2831	0.2485–1.0562
Weight loss before CTH									
Yes	17 (43.6 %)	22 (56.4 %)	0.9567, 1.0245	6	0.7040	0.9058	18	0.0376	0.4632
No	19 (44.2 %)	24 (55.8 %)	0.4278–2.4538	3	0.1443	0.5436–1.5092	7.5	4.3237	0.2242–0.9568
Anemia before CTH									
Yes	32 (54.2 %)	27 (45.8%)	0.0189, 0.3302	3	0.0154	0.5430	11	0.3844	0.7685
No	9 (28.1 %)	23 (71.9 %)	0.1309–0.8329	6.5	5.8656	0.3313–0.8901	13	0.7565	0.4246–1.3910
Side effect after CTH									
Yes	–	–	–	4.5	0.3008	0.7515	12	0.3435	0.7231
No				4	1.0706	0.4374–1.2911	21	0.8974	0.3697–1.4142
Subsequent lines of treatment									
Yes	–	–	–	5	0.7157	0.9131	16.5	0.0305	0.5011
No				3	0.1326	0.5596–1.4897	8	4.6802	0.2680–0.9371
Family history of cancer (any malignant)									
Yes	6 (33.3 %)	12 (66.7 %)	0.3367, 1.7500	3.5	0.8389	0.9349	7.5	0.4320	0.7412
No	21 (46.7 %)	24 (53.3 %)	0.5588–5.4810	3	0.0413	0.4887–1.7885)	11.5	0.6175	0.3511–1.5645
*RRM1* (−37C>A)									
AA	–	5 (100 %)	–	10.5	0.0986	–	18.5	0.0677	–
AC	23 (47.9 %)	25 (52.1 %)		4	4.6330		18	5.3846	
CC	15 (42.9 %)	20 (57.1 %)		3.5			9.5		
AA	–	5 (100 %)	0.1352, 9.3077	10.5	0.0437	2.1736	18.5	0.8528	1.1004
AC or CC	38 (45.7 %)	45 (54.3 %)	0.4986–173.75	3.5	4.0687	1.0222–4.6220	11.5	0.0344	0.4006–3.0223
AC	23 (47.9 %)	25 (52.1 %)	0.3270, 0.6522	44	0.7840	1.0707	18	0.0351	1.9088
AA or CC	15 (37.5 %)	25 (62.5 %)	0.2775–1.533		0.0751	0.6569–1.7452	11	4.4381	1.0460–3.4833
CC	15 (42.9 %)	20 (57.1 %)	0.9601, 1.0222	3.5	0.0928	1.5613	9.5	0.0193	2.1346
AA or AC	23 (43.3 %)	30 (56.7 %)	0.4316–2.4208	5.5	2.8249	0.9286–2.6251	18	5.4786	1.1312–4.0281
*RRM1* (−524C>T)									
CC	1 (14.3 %)	6 (85.7 %)	–	10.5	0.0923	–	18.5	0.4129	–
CT	24 (50 %)	24 (50 %)		3.5	4.7664		11.5	1.7691	
TT	16 (44.4 %)	20 (55.6 %)		3.5			10		
CC	1 (14.3 %)	6 (85.7 %)	0.1237, 5.4545	10.5	0.0343	2.1249	18.5	0.5983	1.2875
CT or TT	40 (47.6 %)	44 (52.4 %)	0.6291–47.293	3.5	4.4805	1.0574–4.2700	11	0.2776	0.5029–3.2959
CT	24 (50 %)	24 (50 %)	0.3174, 0.6538	3.5	0.9141	0.9740	11.5	0.3318	0.7505
CC or TT	17 (39.5 %)	26 (60.5 %)	0.2843–1.504	6	0.0116	0.6035–1.5720	11	0.9417	0.4204–1.3399
TT	25 (45.4 %)	30 (54.6 %)	0.9246, 0.9600	3.5	0.0983	1.5342	10	0.1807	1.5055
CT or CC	16 (44.4 %)	20 (55.6 %)	0.4124–2.2347	4	2.7336	0.9237–2.5484	13	1.7920	0.8270–2.7404

### Overall survival

The median OS in the study population was 12 months. The median OS was significantly longer in patients without weight loss prior to chemotherapy compared to other patients (respectively, 18 vs 7.5 months, *p* = 0.0376; HR = 0.46, 95 % CI 0.22–0.96). Patients who were treated with subsequent lines of treatment showed significantly longer OS than patients who received only one line of treatment (16.5 vs 8 months, *p* = 0.0305; HR = 0.50, 95 % CI 0.27–0.94). However, when compared separately none of second-line scheme has not improve survival significantly (ERL vs other 11.5 vs 10 mo.; *p* = 0.4270, HR = 1.37, 95 % CI 0.63–2.99; PEM vs other 13 vs 11 mo.; *p* = 0.1813, HR = 1.51, 95 % CI 0.82–2.76; DCX vs other 16.5 vs 12 mo.; *p* = 0.3365, HR = 0.67, 95 % CI 0.29–1.52). There was no statistically significant effect of other factors on the OS.

**Fig. 4:**
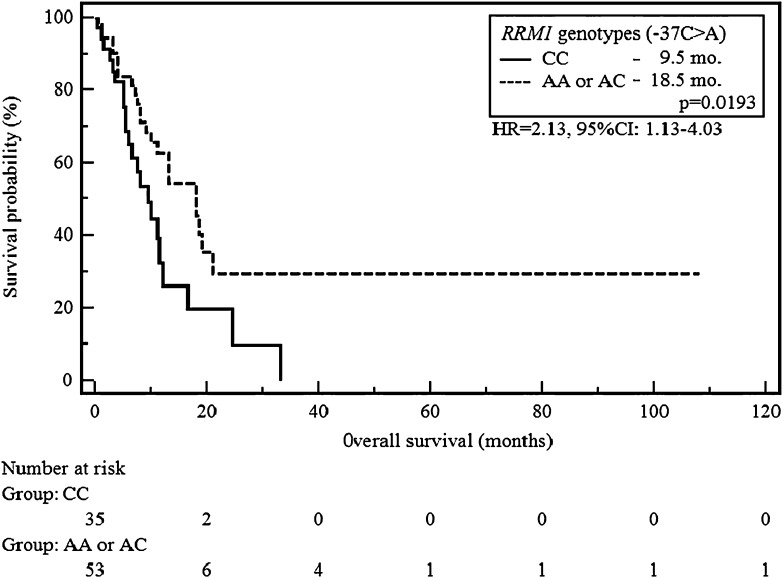
The probability of overall survival change depending on *RRM1* genotype (−37C>A)

**Fig. 5:**
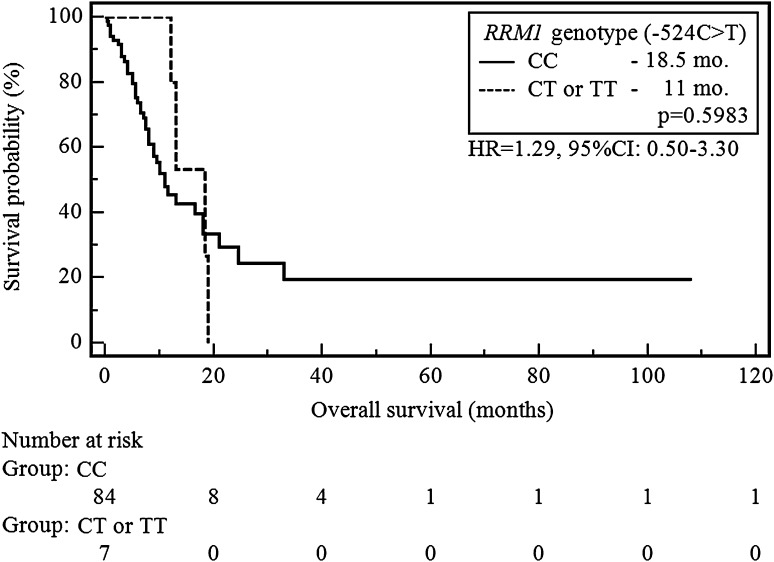
The probability of overall survival change depending on *RRM1* genotype (−524C>T)

The CC genotype carriers of *RRM1* (−37C>A) showed a significant increase in the risk of shortening of OS compared to patients with AA or AC genotypes (9.5 vs 18 months, *p* = 0.0193; HR = 2.13, 95 % CI 1.13–4.03, Fig. 4). None of the SNP −524C>T variants did significantly affect the length of OS in the study group (e.g. CC vs CT or TT, 18.5 vs 11 months, *p* = 0.5983, HR = 1.29, 95 % CI 0.50–3.30; Fig. 5).

Poor performance status (PS = 1, *p* = 0.0053, HR = 5.81, 95 % CI 1.70–19.93) and lack of subsequent lines of treatment (*p* = 0.0246, HR = 2.58, 95 % CI 1.15–7.53) were only factors that shorten OS in a Cox multivariate logistic regression analysis (overall fit of the model *χ*^2^ = 27.73, *p* = 0.0233).

## Discussion

Despite progress in medicine, that has been made in recent years, there are still lack of predictive factors, which would allow qualify NSCLC patients to appropriate chemotherapy regimen. The current criteria of patients’ selection to cytostatic-based therapy are primarily performance status, treatment toxicity and clinician’s experience; however, it seems to be insufficient. In addition, most of the currently available chemotherapy regimens, regardless of the line of treatment, are characterized by significant differences in the effectiveness in various patients. This is another evidence that key to prediction of occurrence of resistance to cytostatics may be stored in genetic information [10–12, 18, 19].

Reliable evaluation of mRNA or protein expression requires access to the tumor tissue Tumor tissue in an advanced stages of NSCLC is difficult or, sometimes, impossible to obtain. Changes in the structure, function, stability, folding or expression of proteins may be caused by occurrence of specific SNPs in encoding or non-coding sequences (especially located in promoter region) of genes. Moreover, the analysis of SNPs may be carried out in materials that are easy to obtain (e.g. DNA from peripheral blood leukocytes) and thus easier introduced into routine clinical practice. Consequently, many studies (unfortunately mainly retrospective) assessed the effect of the individual polymorphic variants of different genes on the effectiveness of various treatment regimens.

There is lack of meta-analysis evaluating the impact of any *RRM1* SNPs on response to treatment, PFS or OS in patients with NSCLC treated with gemcitabine. Among the eight original papers investigating relationship of SNPs (−37C>A and/or −524C>T) with response to treatment, four of them concerned Asian patients. Feng et al. analyzed both mentioned SNPs (genotypes) independently and in combination in 214 patients treated with different schemes chemotherapy based on platinum. Kim et al. evaluated allelotype of two mentioned SNPs in 97 patients received chemotherapy based on gemcitabine. Lin et al. estimated only relationship between −37C>A polymorphism and effectiveness of chemotherapy regimens containing gemcitabine in 40 NSCLC patients. Dong et al. assessed SNPs in 56 patients treated with chemotherapy based on gemcitabine [16, 17, 20, 21]. In studies concerning Caucasian population, analysis of −37C>A SNP was presented by Isla et al. (62 patients received platinum compounds in combination with docetaxel), Vinolas et al. (94 patients treated with platinum compounds in combination with vinorelbine) and Mlak et al. (62 patients treated with platinum compounds in combination with gemcitabine). While, Ludovini et al. assessed −524C>T SNPs in 168 patients received platinum-based chemotherapy [22–25].

Feng and co-workers did not demonstrate the association between the −37C>A SNP and the response to chemotherapy. However, the statistically significant relationship between −524C>T SNP of *RRM1* and response to treatment (*χ*^2^ = 6.179, *p* = 0.046) was proved [16]. In contrast, Kim et al. reported a significantly higher response rate in patients with genotype AC/CT (−37C>A/−524C>T) in comparison to carriers of other combinations of *RRM1* allelotypes (65.5 vs 42.6 %, *p* = 0.039) [18]. Lin et al. and Dong et al. have shown no significant differences in response to treatment depending on the *RRM1* genotype [17, 19]. Isla et al., Vinolas et al., Ludovini et al. demonstrated no correlation between the genotype of *RRM1* and response to the treatment [23–25].

The only evidence for association of −37A>C polymorphism and response to gemcitabine-based chemotherapy was reported in our previous study. We indicated that AC genotype was significantly related with less frequent response to chemotherapy (*χ*^2^ = 5.47, *p* = 0.0193). It was probably caused by the influence of A allele (importance of AA genotype was impossible to assess due to small study group). In addition, this study showed a significant association between the presence of genotype AA or AC and early progression of the disease (*χ*^2^ = 3.61; *p* = 0.0573) [22].

In the available literature six studies describe the relationship between SNPs of *RRM1* and the duration of the PFS (Dong et al., Mlak et al., Vinolas et al., Isla et al., Kim et al., Ludovini et al.) and OS (Ryu et al., Mlak et al., Vinolas et al., Isla et al., Kim et al., Ludovini et al.) in patients with NSCLC treated in the first-line with platinum and third generation drug scheme (usually gemcitabine) [16, 20, 22–26]. Dong et al. presented the positive study regarding the impact of *RRM1* SNPs on the length of PFS. Authors demonstrated significant differences in the PFS depending on −37C>A SNP (23.3, 30.7, 24.7 weeks for Asian patients with CC, CA, AA genotype, respectively, *p* = 0.043) [17]. Among the studies concerning Caucasian patients, only in our previous publication the presence of a significant effect of CC genotype (−37C>A) on reduction of the risk of shortening both PFS (6 months for patients with CC genotype vs 2 months for others patients, HR = 0.51, 95 % CI 0.29–0.89, *p* = 0.0087) and OS (16.5 months for patients with CC and 8 months for patients CA or AA genotypes, HR = 0.47; 95 % CI 0.22–0.99, *p* = 0.0448) was demonstrated [22]. Other studies revealed no significant association between polymorphic variants of *RRM1* (−37C>A and/or −524C>T) and the length of PFS and/or OS in patients with NSCLC treated with first-line chemotherapy.

Therefore, statistically significant relationship between −524C>T SNP of *RRM1* and the PFS length (CC genotype was significantly associated with prolongation of PFS) in patients with advanced NSCLC who were treated first-line chemotherapy based on platinum compounds and gemcitabine was described for the first time in present study. We also verified the results of the impact of −37C>A SNP on the PFS (AA genotype was significantly associated with PFS prolongation) and OS (CC genotype was significantly associated with OS shortening), described previously in smaller populations of NSCLC [17, 22]. The limitations of our research are retrospective character of analysis and heterogeneous study group.

Unfortunately, despite growing evidence suggesting that SNPs in genes encoding proteins involved in drug metabolism and DNA repair may help to explain the inter-individual variability of response or resistance to chemotherapy, most of available studies present conflicting results.

Differences between studies may be due to: (1) race differences (Asian vs Caucasian patients), which are reflected both in the incidence of SNPs and in phenotypic disparity; (2) NSCLC has many subtypes (characterized by, e.g.: differences in driver mutations occurrence and clinical course) thus in most studies different proportion of each subtype may occur. Differences in course of treatment: in some studies; (3) part of patients received chemoradiation. In first-line regimens different platinum compound (cis- or carboplatin) may be used; (4) In subsequent lines most patients are treated with multiple drugs, including: pemetrexed, docetaxel, and an TKI, as well as may undergo surgery, which significantly affects course of disease and survival.

Accordingly, it is crucial to conduct a large randomized prospective studies taking into account the respective proportions of race, subtypes of NSCLC, as well as based on suitable standards and uniform regimens of treatment.

## Conclusions

The presence of rare genotypes: AA (−37C>A) and CC (−524C>T) of *RRM1* promoter are favorable predictors associated with prolongation of PFS in NSCLC patients treated with first-line chemotherapy with platinum compounds and gemcitabine. Moreover, occurrence of CC genotype (−37C>A) is unfavorable predictor of OS shortening. Evaluation of selected SNPs of *RRM1* may in the future, become a useful tool in the qualification of patients with NSCLC to the appropriate chemotherapy regimen. However, our results should be previously confirmed in sufficiently large and prospective studies.

All procedures performed in studies involving human participants were in accordance with the ethical standards of the institutional and/or national research committee and with the 1964 Helsinki Declaration and its later amendments or comparable ethical standards. The study was approved by the Committee Ethics and Research at the Medical University of Lublin (no. consent: KE-0254/142/2010). This article does not contain any studies with animals performed by any of the authors. Informed consent was obtained from all individual participants included in the study.
